# Supplementary material for: CYP7A1, NPC1L1, ABCB1, and CD36 Polymorphisms Associated with Coenzyme Q10 Availability Affect the Subjective Quality of Life Score (SF-36) after Long-Term CoQ10 Supplementation in Women
Source: Nutrients. 2022 Jun 22;14(13):2579. doi: 10.3390/nu14132579 (PMC9268390; doi:10.3390/nu14132579)
Supplement: Supplementary file 1 [file nutrients-14-02579-s001.zip › Figure S1-S2_20220608.pptx]

## Slide 1
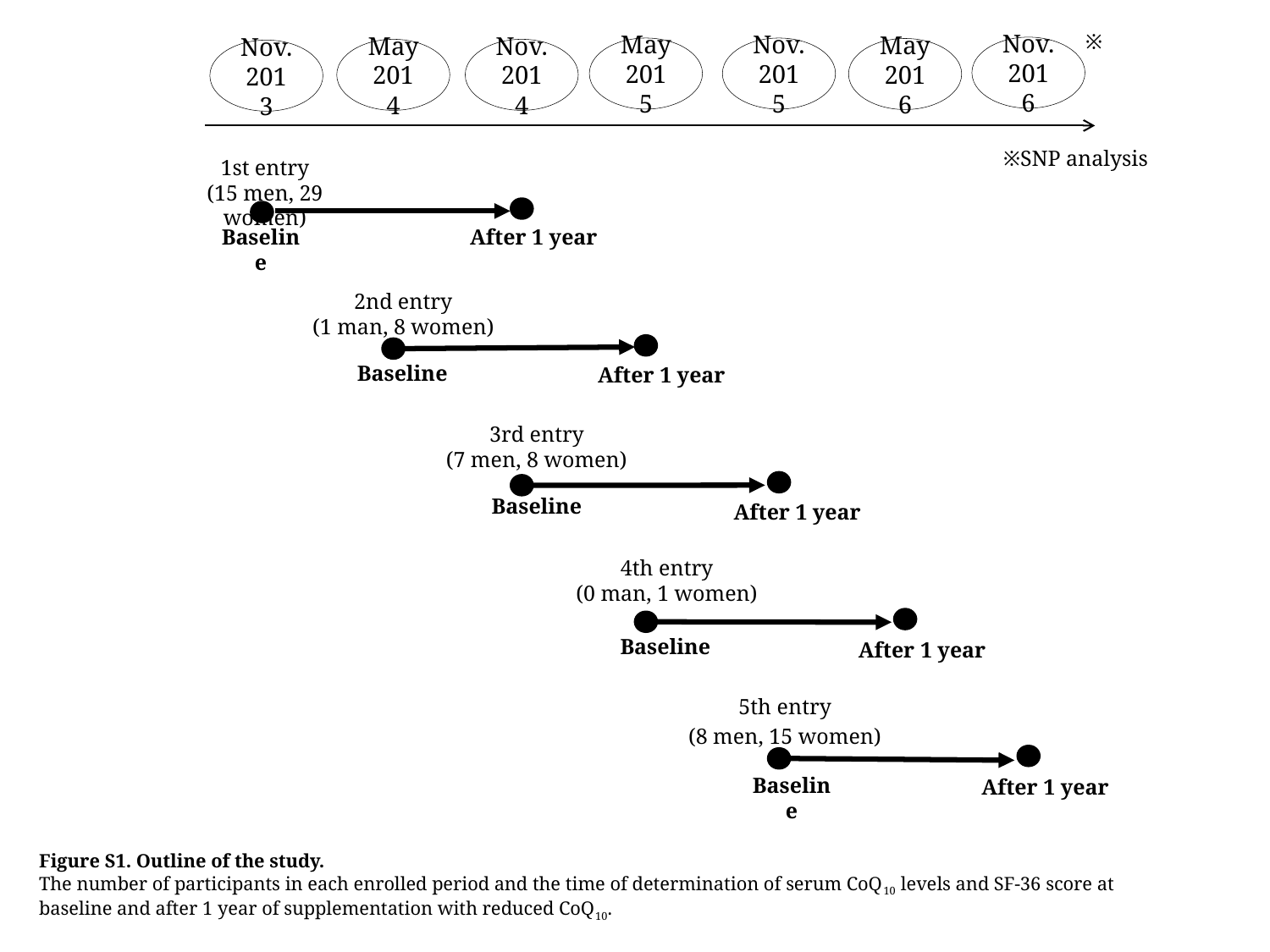

※
Nov.
2016
May
2015
Nov.
2015
May
2016
May
2014
Nov.
2014
Nov.
2013
※SNP analysis
1st entry
(15 men, 29 women)
Baseline
After 1 year
2nd entry
(1 man, 8 women)
Baseline
After 1 year
3rd entry
(7 men, 8 women)
Baseline
After 1 year
4th entry
(0 man, 1 women)
Baseline
After 1 year
5th entry
(8 men, 15 women)
Baseline
After 1 year
Figure S1. Outline of the study.
The number of participants in each enrolled period and the time of determination of serum CoQ10 levels and SF-36 score at baseline and after 1 year of supplementation with reduced CoQ10.

## Slide 2
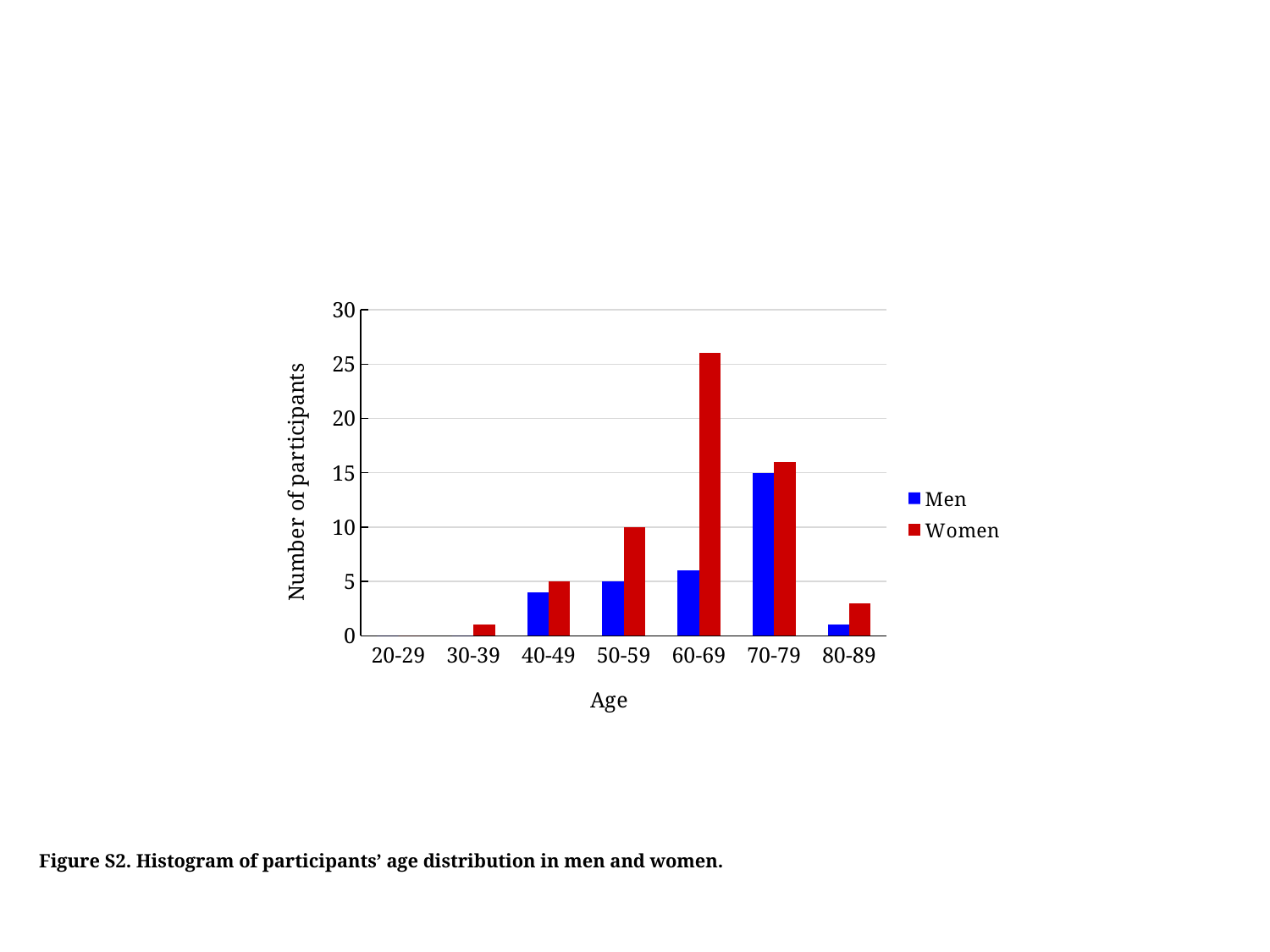

### Chart
| Category | Men | Women |
|---|---|---|
| 20-29 | 0.0 | 0.0 |
| 30-39 | 0.0 | 1.0 |
| 40-49 | 4.0 | 5.0 |
| 50-59 | 5.0 | 10.0 |
| 60-69 | 6.0 | 26.0 |
| 70-79 | 15.0 | 16.0 |
| 80-89 | 1.0 | 3.0 |Figure S2. Histogram of participants’ age distribution in men and women.
